# Surfactant‐Assisted Construction of Covalent Organic Frameworks

**DOI:** 10.1002/advs.202501580

**Published:** 2025-04-27

**Authors:** Youqi Li, Qingqing Zhang, Zhendong Dai, Renzhong Wang, Zhaohua Li, Yu Huang, Runchen Lai, Facai Wei, Feng Shao

**Affiliations:** ^1^ State Key Laboratory of Materials‐Oriented Chemical Engineering College of Chemical Engineering Nanjing Tech University Nanjing 211816 China; ^2^ Suzhou Laboratory Suzhou 215100 China; ^3^ School of Materials Science and Engineering Suzhou University of Science and Technology Suzhou 215109 China

**Keywords:** covalent organic frameworks, crystallinity, dispersion and processability, morphology, surfactant‐assisted synthesis

## Abstract

Covalent organic frameworks (COFs), characterized by their unique ordered pore structures, chemical diversity, and high degree of designability, have demonstrated immense application potential across multiple fields. However, traditional synthesis methods often encounter challenges such as low crystallinity and uneven morphology. The introduction of surfactants has opened up new pathways for the synthesis of COFs. Leveraging their intermolecular interactions and self‐assembly properties, surfactants can effectively regulate the nucleation, growth processes, and ultimate structure and properties of COFs. This paper systematically reviews the latest research achievements and future trends in surfactant‐assisted COF synthesis, emphasizing the crucial role of surfactants as key additives in the preparation of COFs. Surfactants not only facilitate uniform nucleation and growth of COFs, enhancing the crystallinity and structural order of the products but also enable precise and diverse regulation of the dimensionality, morphology, and structure of COFs. Furthermore, by influencing the dispersion and processability of COFs, surfactants enhance their practicality and workability. Finally, the paper presents some prospects for the challenges and future opportunities in this emerging research area.

## Graphical abstract

This paper presents a comprehensive overview of the latest advancements and future trends in the surfactant‐assisted synthesis of covalent organic frameworks. It emphasizes the crucial role of surfactants as key additives in optimizing the crystallinity, morphology, and structure of COFs, facilitating diverse regulation, and enhancing their practical applications.

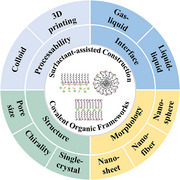

## 1 Introduction

Covalent organic frameworks (COFs) represent an emerging class of crystalline porous organic polymers assembled from organic molecular precursors through reversible covalent bonds and ordered stacking, first reported by Yaghi et al. in 2005.^[^ ^1^ ^]^ The core construction mechanism of COFs relies on dynamic covalent bond‐mediated condensation reactions, thereby establishing stable framework structures.^[^ ^2^ ^]^ The COFs generally exhibit robust molecular frameworks, exceptional structural regularity, highly ordered pore size distributions, and inherent high porosity along with designable chemical stability.^[^ ^3^, ^4^ ^]^ Furthermore, their surfaces are enriched with various active sites, offering abundant functionalization possibilities within the field of materials science. Thanks to their multifunctionality, flexibility in topological structures, and diversity in linkage modes, COFs have emerged as a powerful platform for molecular design and functional customization of organic materials.^[^ ^5^ ^]^ They are widely applied in various cutting‐edge fields such as catalytic reactions, molecular separation, energy storage, and gas/liquid adsorption, demonstrating extensive application potential and promising development prospects.^[^ ^6^, ^7^ ^]^

To date, in the pursuit of preparing COFs with intricate structures and diverse functionalities, researchers have explored and developed a multitude of synthetic strategies. These encompass, but are not limited to, solvothermal synthesis, sonochemical synthesis, microwave‐assisted synthesis, and mechanochemical synthesis.^[^ ^8^, ^9^ ^]^ Among these, solvothermal synthesis stands as a mainstream technique in molecular design and crystal engineering, having successfully constructed thousands of COF materials, thereby demonstrating its formidable construction capability.^[^ ^10^ ^]^ However, traditional solvothermal synthesis often necessitates stringent reaction conditions, such as the use of harmful organic solvents, high‐temperature and inert gas environments, and lengthy reaction durations. Furthermore, it requires complex operational procedures, including freeze‐pump‐thaw cycles and precise sealing of pipelines, posing stringent demands on experimental conditions.^[^ ^11^ ^]^ Additionally, COFs synthesized via solvothermal methods typically exist as insoluble and non‐melting powders, presenting challenges in precisely controlling their crystallite domain sizes and morphologies. This, in turn, hinders the progress of COF materials in scale‐up production, customized functional development, processing, and commercial applications. To address these limitations, a series of novel, gentler, and more environmentally friendly synthetic methods have emerged in recent years, exhibiting significant advantages in enhancing synthetic efficiency and facilitating scale‐up production.^[^ ^12^, ^13^ ^]^ Despite the rapid advancements, achieving morphological presetting and precise control of COF materials within these novel methods remains one of the pivotal issues urgently needing resolution in current research. Therefore, the continuous exploration and optimization of COF synthesis strategies are of paramount importance for facilitating their transition from laboratory research to practical applications.

Surfactants, as a unique class of organic compounds, are characterized by their molecular structures that integrate polar hydrophilic moieties with nonpolar hydrophobic groups. This unique characteristic imparts them with the ability to significantly reduce interfacial or surface tension. In recent years, the application scope of surfactants has far surpassed traditional domains, particularly exhibiting remarkable potential in the synthesis of metal‐organic frameworks (MOFs) as emulsifiers, foaming agents, surface modifiers, structure‐directing agents, and dispersants.^[^ ^14^, ^15^ ^]^ These roles have facilitated the creation of MOFs with diverse sizes, morphologies, and crystalline phases.^[^ ^16^, ^17^ ^]^ Currently, this innovative approach is gradually being extended to the synthesis of COFs. Although preliminary indications of this trend can be found in the literature, a comprehensive and systematic review is lacking. There is an urgent need to integrate and analyze the latest research findings, mechanisms of action, challenges faced, and future opportunities associated with the use of surfactants in COFs synthesis.

**Figure 1:**
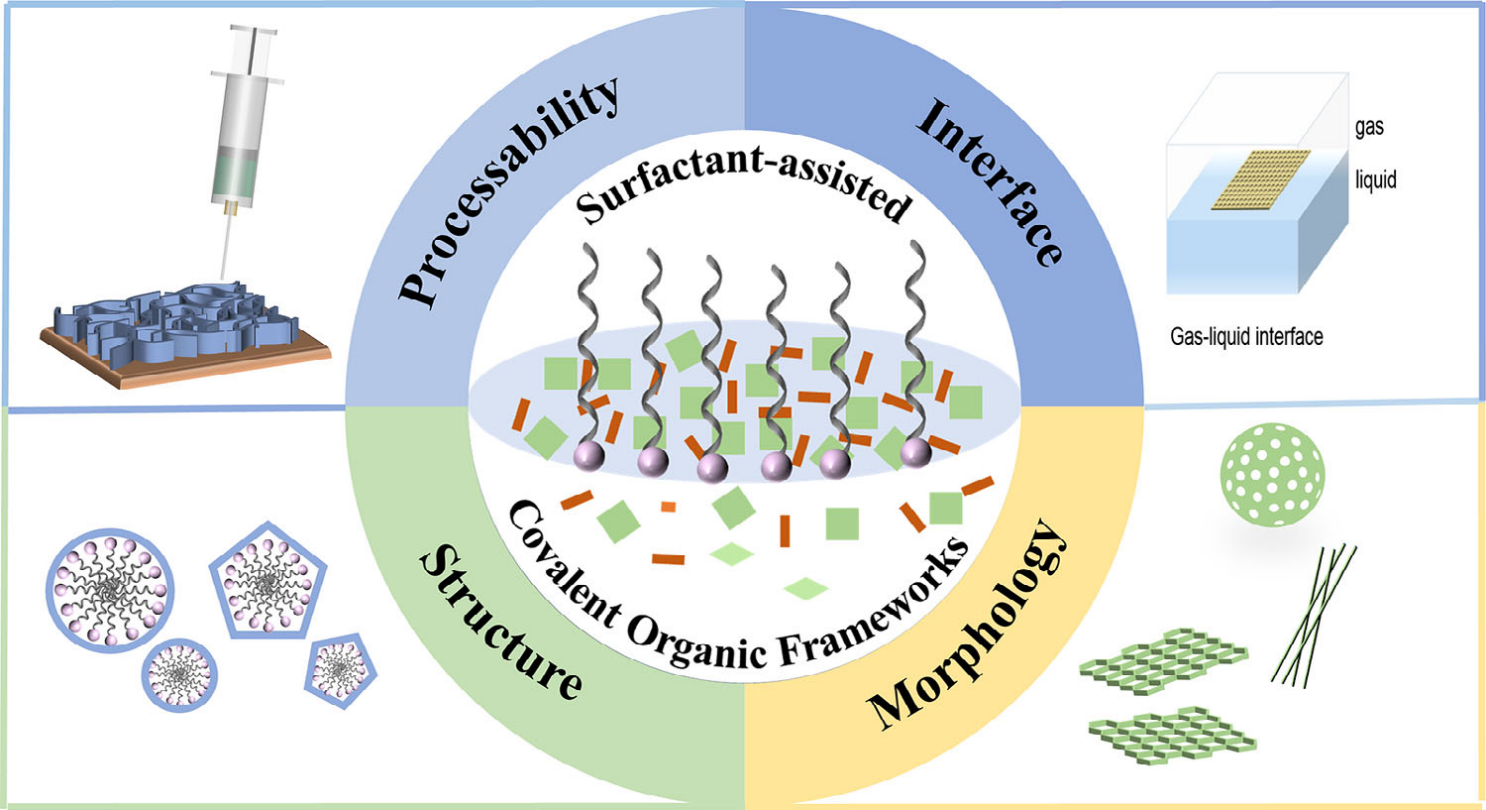
Overview diagram of surfactant‐assisted construction of COFs.

In light of this, the present paper aims to address this knowledge gap by reviewing the typical cases and significant breakthroughs in surfactant‐assisted synthesis of COFs in recent years (**Figure** **1**). It delves into how surfactants function as crucial mediators in precisely regulating the crystallographic structure, morphology, and processing properties of COF products, while also elucidating the underlying mechanisms. Furthermore, this paper focuses on the challenges faced by surfactants in the field of COF growth, including optimization of reaction conditions, enhancement of product purity, and scale‐up production. Additionally, it provides insights into the future directions and potential applications of this field. This paper not only represents a systematic overview of surfactant‐assisted synthesis strategies and chemical methods for COFs but also serves as a valuable reference framework and source of inspiration for scholars interested in exploring this emerging research area. It aims to foster continuous prosperity and innovation within this domain.

## 2 Surfactant‐Assisted Construction of COFs

### 2.1 Interfacial Preparation of COF Films

Interface‐assisted synthesis leverages interfacial effects to facilitate the direct assembly of monomeric building blocks into COF films.^[^ ^18^ ^]^ Unlike the homogeneous growth in a single‐phase synthesis system that typically yields amorphous powders, the introduction of an interface within the synthetic setup provides a flat and uniform surface for 2D confinement.^[^ ^19^ ^]^ This allows for further control over the arrangement and self‐assembly of monomers under interfacial conditions, guiding their lateral growth in the 2D direction.^[^ ^20^ ^]^ Consequently, large‐area films with single‐layer, few‐layer, or controlled thicknesses can be produced on a large scale. Furthermore, compared to reactions occurring in homogeneous media, reactions confined at interfaces often exhibit higher reactivity and yield.^[^ ^21^ ^]^ Therefore, interface‐assisted synthesis has gradually evolved into a commonly used method for efficiently constructing COF films.

#### 2.1.1 Gas–Liquid

The air‐water interface, as one of the most ubiquitous and easily established gas‐liquid interfaces in nature, spontaneously generates an extremely smooth interfacial region with a surface roughness as low as 3 Å.^[^ ^22^ ^]^ This characteristic provides an ideal venue for the 2D confined polymerization of COF monomers while ensuring a high migration rate of the monomers, facilitating subsequent easy transfer of the film structure.^[^ ^23^ ^]^ However, the random orientation and extensive movement of monomers at the interface, coupled with the absence of supramolecular driving forces to promote pre‐organization into long‐range ordered structures, often result in COF films with poor crystallinity and disorganized lamellar orientations.^[^ ^24^ ^]^ Notably, the surface tension at gas‐liquid interfaces plays a pivotal role in governing molecular assembly dynamics. Higher surface tension tends to induce disordered aggregation of monomers through excessive lateral compression, whereas lower surface tension may compromise structural integrity due to insufficient molecular packing density.^[^ ^25^ ^]^

To address this issue, surfactants have been introduced. Surfactants effectively mediate this balance by forming ordered interfacial templates. Their amphiphilic nature enables spontaneous adsorption at the interface, creating dynamic molecular gradients that regulate both vertical compression and lateral diffusion of monomers.^[^ ^24^ ^]^ This surface tension modulation not only reduces interfacial free energy but also establishes thermodynamic conditions conducive to ordered assembly.^[^ ^26^ ^]^ In recent years, surfactants have been extensively utilized to assist in the construction of rigid and highly crystalline COFs at the water/air interface.^[^ ^27^ ^]^ They spontaneously assemble into stable structures at the interface, and these ordered and closely packed arrangements effectively control the anisotropic growth process of 2D COFs, thereby aiding in the synthesis of highly crystalline and functionalized 2D COF materials. This strategy typically encompasses the following three key steps: First, surfactant molecules are uniformly dispersed and spread across the water surface to form a stable, organized monomolecular floating layer with consistent surface pressure. During this process, the selection of surfactants should be based on potential interactions with monomer precursors, including electrostatic interactions, coordination bonds, hydrogen bonds, and strong covalent bonds, depending on the functional groups present in the monomers.^[^ ^28^ ^]^ Second, a dispersion of monomer 1 is injected into the system, allowing it to diffuse, adsorb beneath the surfactant monolayer, and undergo pre‐organization.^[^ ^29^ ^]^ Finally, monomer 2 is introduced onto the water surface to trigger the polymerization reaction.^[^ ^30^ ^]^ By finely tuning the monomer concentration and reaction time, the thickness of the resulting COF films can be flexibly adjusted within the range of 1 to 100 nanometers.

**Figure 2:**
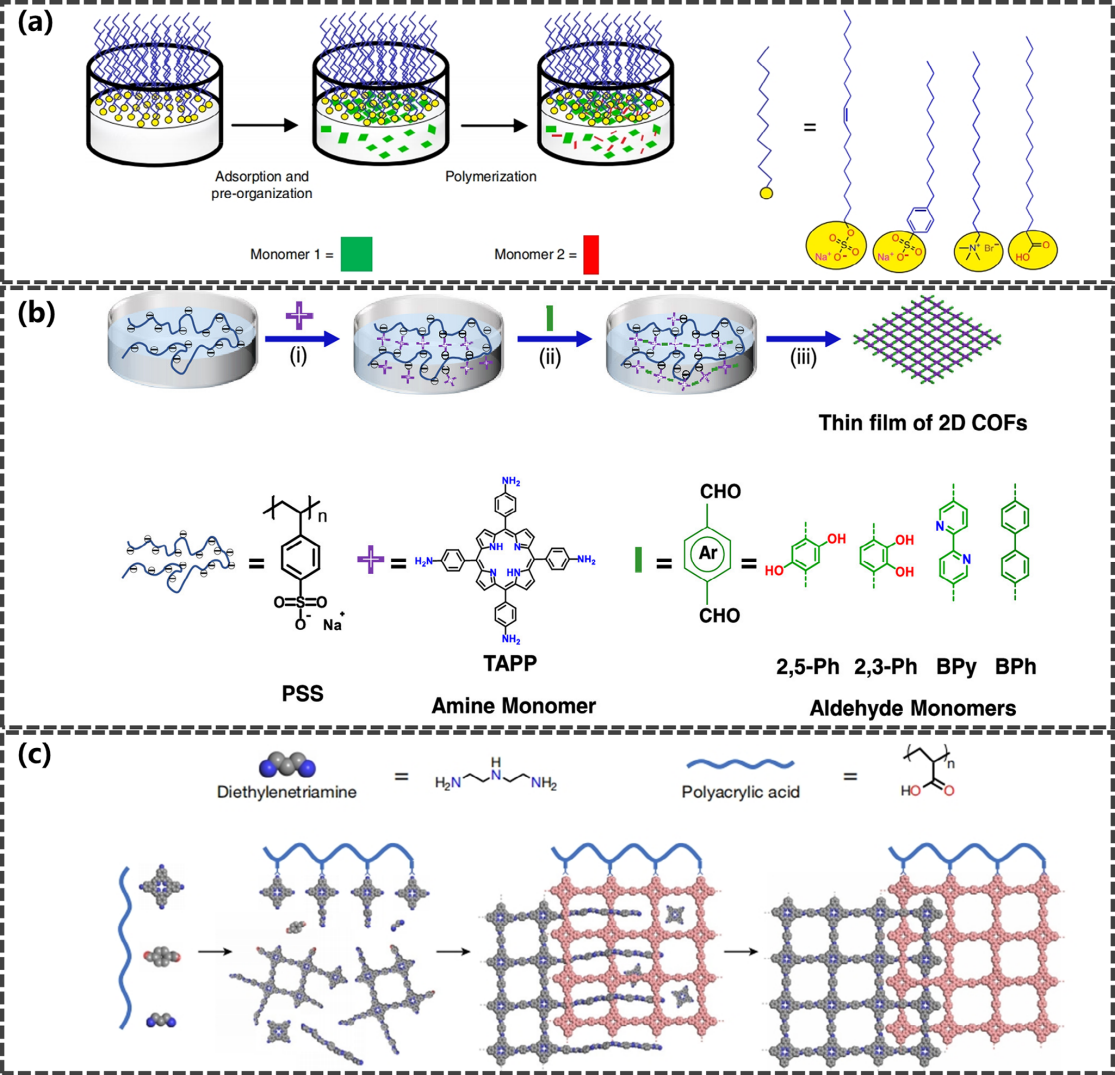
Schematic illustration depicting the synthesis strategies for the preparation of 2D COFs membranes at the air‐water interface, mediated by a) SOS, Reproduced with permission.^[^ ^31^ ^]^ Copyright 2019, Springer Nature. b) PSS, Reproduced with permission.^[^ ^32^ ^]^ Copyright 2022, American Chemical Society. and c) polyacrylic acid, respectively. Reproduced with permission.^[^ ^33^ ^]^ Copyright 2024, Springer Nature.

As the first illustrative example, Feng et al., building upon their previous synthesis of quasi 2D crystalline conductive polymer polyaniline films,^[^ ^34^ ^]^ introduced the surfactant monolayer ‐assisted interfacial synthesis technique for the first time, achieving few‐layer 2D COFs with high crystallinity and crystal sizes of several micrometers (**Figure** **2**a).^[^ ^31^ ^]^ Specifically, with the assistance of the surfactant sodium oleyl sulfate (SOS), various ultra‐thin (≈2 nm, equivalent to roughly 5 layers) polyimide crystals with an average crystal domain size of ≈3.5 µm^2^ were synthesized at the water surface through the reaction between anhydride and amine monomers. By meticulously selecting various surfactants, they validated the formation mechanism of the COF films, which was attributed to the pre‐ordered arrangement of monomers at the water‐surfactant interface, thereby facilitating the formation of highly crystalline 2D COF films. Notably, the binding motif between surfactant headgroups and monomers dictates molecular orientation at the interface. Carboxylate‐containing surfactants (e.g., stearic acid) form covalent amide bonds with amine moieties, enforcing edge‐on monomer alignment whereas sulfonate surfactants (e.g., SOS) promote face‐on stacking through weaker electrostatic steering. This geometric control directly determines the crystallographic growth direction—vertical polymerization for edge‐on versus horizontal propagation for face‐on configurations. These findings establish that surfactant chemistry governs not just the crystallization degree but fundamentally rewires the hierarchical assembly pathway—from molecular orientation to mesoscale stacking architecture. The ability to program growth directions (horizontal→vertical) while enhancing crystallinity demonstrates the dual role of surfactants as structural directors and kinetic modulators in COF film engineering.

Additionally, they prepared crystalline thin‐layer polyamide materials with a dual‐pore lattice structure and a crystal domain area of ≈0.3 µm^2^. Subsequently, they utilized aberration‐corrected high‐resolution transmission electron microscopy(AC‐HRTEM) with an ultra‐high resolution of 2.3 Å to directly observe the grain boundaries of another 2D polyimide with a layered stacking structure obtained through a similar method, providing deep insights into its formation mechanism.^[^ ^35^ ^]^ They discovered that, under the influence of surfactants, the growth of polyimide followed a unique “birth and spread ” mode, in which the anti‐phase boundaries could self‐correct missing connections and node defects, forming tilted boundaries through the coalescence of grains. Combined with quantum mechanical calculations, they further confirmed that this boundary reconstruction was energetically feasible and that the mechanism could be extended to other 2D polymer systems. Furthermore, they elaborated on a step‐by‐step molecular‐level mechanism through the combination of in situ techniques and theoretical calculations, emphasizing the crucial role of charged surfactant monolayers in epitaxially guiding the pre‐ordered arrangement of reactant molecules.^[^ ^36^ ^]^ The negatively charged surfactant monolayers on the water surface electrostatically guided the epitaxial arrangement and assembly of amino‐substituted porphyrin molecules, forming well‐defined J‐aggregate structures. This constrained geometry facilitated the subsequent oriented arrangement of perylene tetracarboxylic dianhydride reagents and selectively formed unilateral imine bonds between the porphyrin and perylene tetracarboxylic dianhydride reagents, ultimately synthesizing various periodically ordered charged 2D COF films on the water surface. This series of studies not only deepened the fundamental understanding of surface chemistry on water but also provided new directions for the synthesis of 2D framework materials at the gas/liquid interface.

The SOS surfactant‐monolayer‐assisted interfacial synthesis method for synthesizing high‐crystallinity 2D COF thin films demonstrates a certain degree of universality. On this basis, in recent years, Feng's groups and their co‐workers have utilized a range of synthetic chemistries, including dynamic covalent reactions (e.g., boronic ester formation,^[^ ^37^ ^]^ Schiff base condensation,^[^ ^38^ ^]^ and condensation reactions,^[^ ^31^ ^]^ as well as dynamic irreversible reactions (such as the Katritzky reaction^[^ ^39^ ^]^ and the Knoevenagel reaction,^[^ ^40^ ^]^ to prepare large‐area 2D COF thin films with different pore sizes and configurations. They have also developed applications for these films in various fields, such as fully crystalline viologen‐immobilized COF thin films for electrochromic devices;^[^ ^41^ ^]^ imine‐based COF films,^[^ ^30^, ^42^ ^]^ boronic ester based 2D COFs,^[^ ^43^ ^]^ for separation; ultrathin 2D polyimide COF film,^[^ ^44^ ^]^ crystalline boronic ester based 2D COFs thin films for field‐effect transistors,^[^ ^37^ ^]^ and positively charged 2D COF film for batteries,^[^ ^45^ ^]^ among others.

In addition to the widely utilized SOS, various other surfactants have been progressively developed for the monolayer‐assisted interfacial synthesis of COF thin films. Qiao et al. employed sodium dodecylbenzene sulfonate (SDBS) as a surfactant and utilized a method similar to the air‐water interface approach to fabricate a series of large‐area, customizable‐shape COF nanofilms.^[^ ^46^, ^47^, ^48^ ^]^ Furthermore, the Zheng et al. has conducted a series of notable studies in this field.^[^ ^49^ ^]^ For instance, they spread sodium dodecyl sulfate (SDS) on the air‐water interface, which facilitated the adsorption of monomers beneath the SDS layer through hydrogen bonding and electrostatic interactions (Figure 2b), leading to subsequent pre‐assembly and crystallization, and ultimately yielding micrometer‐sized single‐crystalline COF thin films.^[^ ^50^ ^]^ Similarly, sodium hexadecyl sulfate)‐mediated synthesis can also yield COFs membranes with domain sizes of several hundred nanometers, while COFs membranes obtained through mediation by other surfactants exhibit significantly lower crystallinity. The length of the hydrophobic chain and the polar groups play crucial regulatory roles in the crystallization at the surfactant‐water interface. In another work,^[^ ^27^ ^]^ they delved deeper into the impact of sulfonate‐based surfactants with varying alkyl chain lengths (dodecyl, tetradecyl, hexadecyl, and octadecyl series) on the crystallization characteristics of imine‐linked COF thin films. Specifically, they observed an enhancement in the crystallization degree of COF thin films as the alkyl chain length of the surfactant gradually increased. Additionally, they also developed a method for synthesizing 2D continuous thin films with large single‐crystalline domains using amphiphilic glycine derivatives on the water surface.^[^ ^49^ ^]^ When tetradecanoyl glycine (C_14_‐Glya) was spread on the water surface, it self‐assembled into an ordered structure with complete surface coverage. Moreover, in 2022,^[^ ^32^ ^]^ they innovatively proposed a general strategy for creating COF thin films with large single‐crystalline domains on the water surface, assisted by the charged polymer sodium polystyrene sulfonate (PSS). During the synthesis process, the negatively charged PSS polymer was spread on the air‐water interface, guiding the assembly/diffusion of protonated monomers, as well as polymerization and crystallization, ultimately forming four types of 2D COF thin films with large single‐crystalline domains.

Despite the successful fabrication of polycrystalline films on water surfaces using synthetic strategies assisted by small‐molecule surfactants or linear polymers, the pursuit of high crystallinity in COF films often engenders intrinsic trade‐offs with mechanical performance. While enhanced crystallinity improves charge transport and structural stability, it may compromise toughness through reduced energy dissipation pathways and/or stress concentration at grain boundaries.^[^ ^51^ ^]^ Notably, the surfactant‐mediated stacking architecture provides counterstrategies. The surfactant‐derived interlayer spacers create nano‐confined regions that enable controlled layer slippage under stress while maintaining crystallographic order. This biomimetic “brick‐and‐mortar” structure combines crystalline domains (bricks) with dynamic interfacial regions (mortar), achieving simultaneous enhancement of toughness and crystallinity. Recently, Zheng et al.^[^ ^33^ ^]^ introduced a groundbreaking approach by selecting polyacrylic acid as an auxiliary polymeric surfactant for interfacial synthesis and ingeniously incorporating a sacrificial small‐molecule structure‐directing agent, aliphatic diamines (Figure 2c). This directing agent effectively promotes the interweaving of crystalline polymer chains between adjacent crystalline domains, thereby enhancing the intertwining of COFs in the grain boundary regions, which is subsequently stabilized through node substitution. This innovative method enables the construction of 2D, high‐crystallinity, imine‐linked polymeric thin films with high mechanical strength, elasticity, and toughness on water surfaces. The thin films maintain a high degree of overall crystallinity over scales of several square centimeters. By employing the strategy of imine‐linked 2D COFs, the authors successfully integrated the structural features and properties of amorphous materials into crystalline materials. This discovery suggests the potential for the structural characteristics of amorphous materials to be widely applicable to other crystalline systems, imparting novel properties to these materials, enhancing their specific applications, and laying a solid foundation for exploring entirely new application areas.

#### 2.1.2 Liquid–Liquid

**Figure 3:**
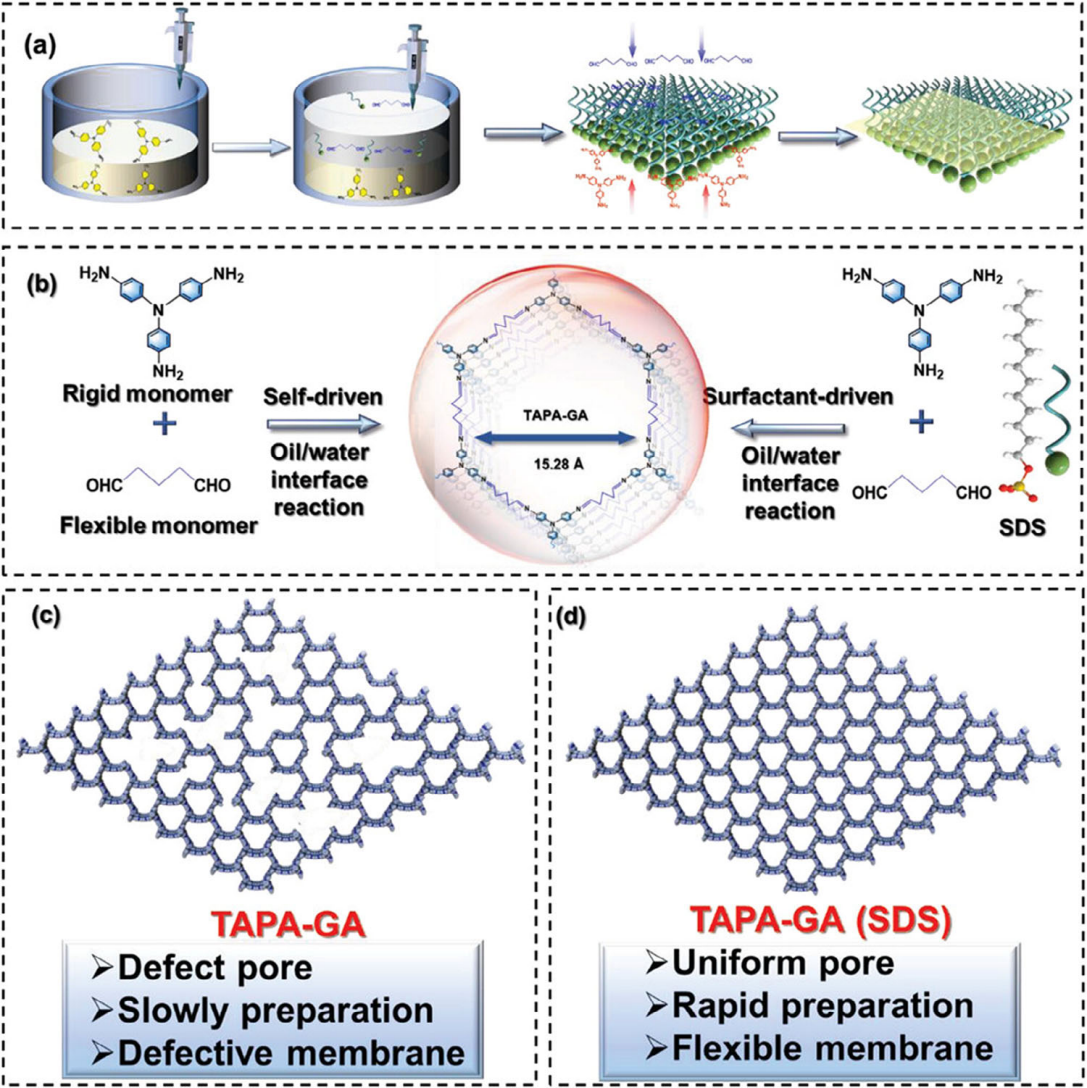
a) SCOF membranes prepared via interfacial reaction, facilitated by SDS as a molecular bridge. b) Synthesis of TAPA‐GA SCOF membranes through condensation with/without SDS. c) TAPA‐GA membrane (without SDS) exhibits heterogeneous pore size distribution. d) TAPA‐GA (SDS) membrane shows uniform pore size distribution. Reproduced with permission.^[^ ^55^ ^]^ Copyright 2023, Wiley‐VCH.

Beyond the common gas‐liquid interface, at the liquid‐liquid interface, the formation kinetics of COFs may differ from those in the bulk phase or gas‐liquid interface due to spatial constraints and interfacial effects.^[^ ^52^, ^53^, ^54^ ^]^ The presence of surfactants can further influence reaction rates and pathways by modulating intermolecular interactions and the properties of the reaction medium, thereby optimizing the crystallinity and pore structure of COFs. Liu et al.^[^ ^55^ ^]^ demonstrated an innovative strategy by carefully selecting aldehyde flexible linkers and triangular building blocks (**Figure** **3**). Under ambient conditions, they successfully utilized SDS‐induced molecular channels in a water/dichloromethane biphasic system to achieve rapid interfacial assembly (completed within ≈5 min) of 2D soft COF (SCOF) films. This achievement marked a new record for the formation rate of SCOF films, with a 72‐fold increase in speed compared to previously reported literature. The research team employed a combination of density functional theory calculations and molecular dynamics simulations to reveal the underlying mechanism of this efficient process. The analysis revealed that the dynamic self‐assembled channels formed by SDS molecules at the liquid‐liquid interface significantly facilitated the efficient transport and uniform distribution of amine precursor molecules in the reaction system, which was crucial for the formation of self‐supporting crystalline imine‐based 2D COF films with high flexibility and structural stability. The prepared COF films not only exhibited an impressive area (reaching 226.9 cm^2^) but also possessed extremely uniform pore size distribution characteristics, opening up new possibilities for applications in separation, catalysis, and energy storage.

Furthermore, at the liquid‐liquid interface, surfactants can significantly reduce the surface tension between two immiscible liquids, thereby stabilizing the interface and preventing droplet coalescence.^[^ ^56^ ^]^ This interfacial stabilization provides an ordered and controllable environment for the dispersion and reaction of COF precursors. The hydrophilic head and hydrophobic tail of surfactants can interact with the two liquids respectively, ensuring uniform distribution of COF precursors at the interface. Wang et al.^[^ ^57^ ^]^ proposed a monomer pre‐assembly process using a surfactant‐assisted interfacial polymerization strategy to prepare high‐crystallinity COF films. Amphiphilic surfactant chains that self‐assemble across the interface can aggregate at the oil‐water interface and pre‐assemble with monomers, improving their interphase transport for achieving complete topological growth. In addition to shortening the polymerization time from 72 to 48 h, the crystallinity and pore uniformity of the COF films were greatly improved. The universality of this strategy was verified by changing the types of monomers and surfactants. Amphiphilic molecule‐assisted interfacial engineering may represent a scalable technique for manufacturing high‐crystallinity framework materials for molecular separation.

### 2.2 Regulating Morphology of COFs

In the pursuit of sustainable and environmentally friendly pathways for the preparation of COFs, the development of green synthesis methods that enable precise morphological control is of particular importance and value.^[^ ^58^, ^59^ ^]^ Surfactants, capable of forming micelles or microemulsion droplets in solution, serve as templates for the growth of COFs, thereby allowing for the regulation of their morphologies.^[^ ^60^ ^]^ By adjusting the type, concentration, and reaction conditions of surfactants, COF materials with diverse morphologies can be synthesized. These different morphologies may impart distinct properties to the COFs, such as specific surface area, porosity, and mechanical strength.^[^ ^61^ ^]^ Furthermore, in biphasic water‐organic solvent systems, surfactants can function as phase‐transfer catalysts, facilitating the transfer and reaction of monomers between the two phases. This aids in the rapid synthesis of COF materials under mild conditions. However, morphological control of COFs remains challenging due to the incompatibility between the self‐assembly of surfactants and the crystallization process of organic phase precursors, and the mechanisms of formation and evolution are not yet well understood.^[^ ^62^ ^]^ From a mechanistic perspective, the resultant morphologies (e.g., nanospheres, nanosheets, or nanofibers) may be governed by surfactant‐mediated interfacial thermodynamics and kinetic pathways.^[^ ^63^ ^]^ In other cases, spherical nanostructures typically may arise from curvature‐dominated assembly, where surfactant micelles impose isotropic constraints on COF nucleation. Lamellar architectures may emerge through planar confinement effects, driven by the anisotropic distribution of surfactant molecules at liquid‐liquid interfaces. Fibrous morphologies may originate from uniaxial alignment under shear stress fields, where surfactant bilayers act as directional scaffolds. The aspect ratio correlates with the persistence length of supramolecular aggregates, modulated by surfactant chain rigidity and solvent polarity. This morphology‐dependent behavior stems from the hierarchical interplay between molecular‐scale interactions (e.g., hydrogen bonding, electrostatic screening) and mesoscale self‐assembly kinetics.^[^ ^17^ ^]^

#### 2.2.1 Nanosheets/Nanoribbons/Nanodisks

**Figure 4:**
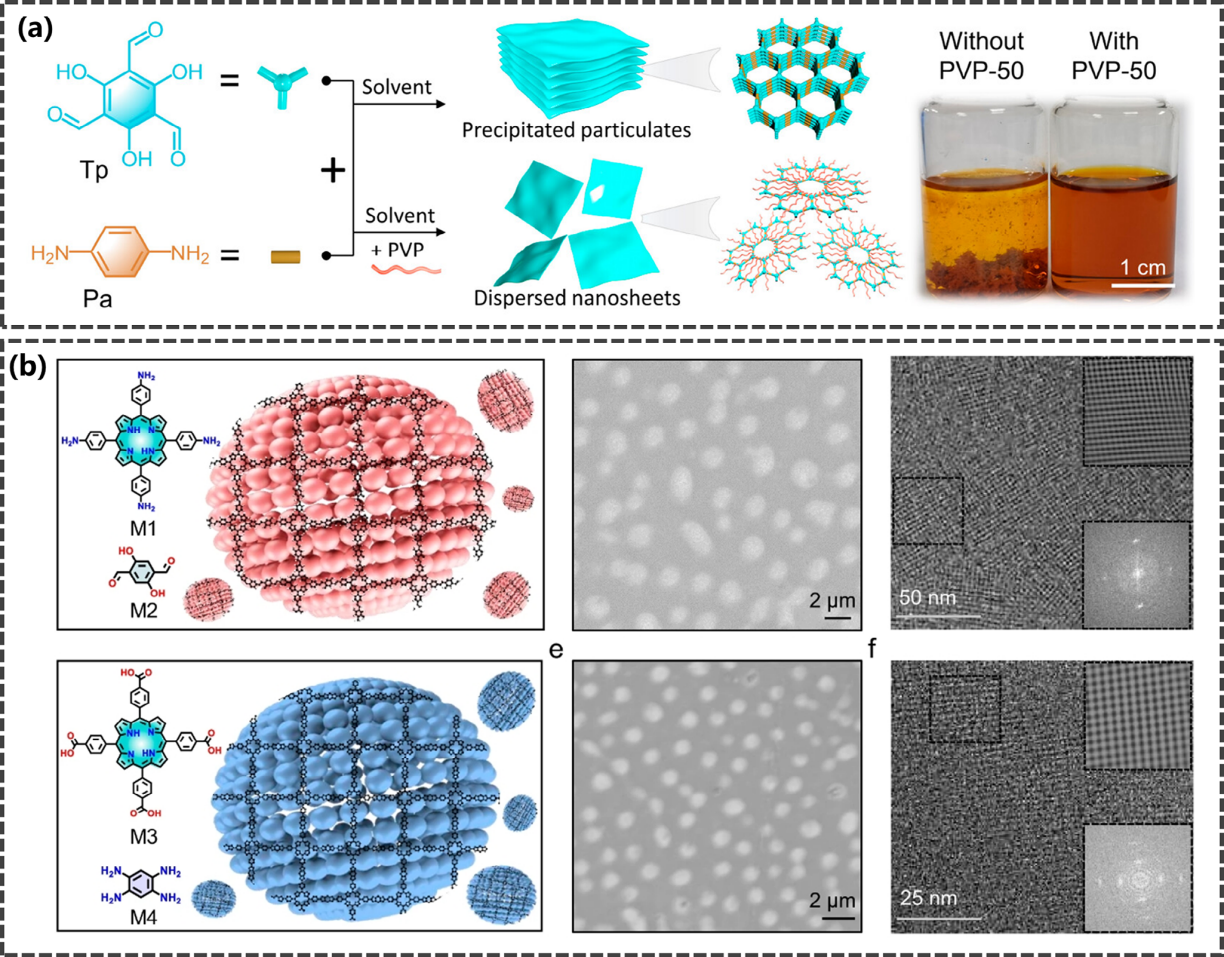
a) Schematic illustration of the synthetic pathway utilizing PVP‐50 as an auxiliary agent for the fabrication of TpPa nanosheets and optical image of the resultant product. Reproduced with permission.^[^ ^66^ ^]^ Copyright 2023, American Chemical Society. b) Schematic diagram and morphological structure characterization of anionic micelle synthesis for 2DPI (top) and 2DPBI (bottom). Reproduced with permission.^[^ ^70^ ^]^ Copyright 2024, Springer Nature.

The hydrophobic long‐chain tails of surfactants can inhibit the interlayer growth of COFs, leading to anisotropic growth of COFs. This inhibitory effect aids in the formation of ultrathin COF nanosheets, enhancing their specific surface area and porosity. Polyvinylpyrrolidone (PVP), as a widely used structural modifier, exhibits significant advantages in controllable preparation, precise thickness control, and easy removal without residue.^[^ ^64^, ^65^ ^]^ It effectively promotes the non‐vertical stacking growth pathway of large‐sized nanosheets during crystallization. Recently, Wang et al.^[^ ^66^ ^]^ proposed a PVP‐induced planar crystallization strategy based on chemical asymmetry, enabling the preparation of highly dispersed, ultrathin COF nanosheets with precisely controllable thickness (**Figure** **4**a). In this strategy, PVP serves as a key regulatory molecule, guiding the anisotropic growth of the COF structure by selectively interacting with aldehyde groups and the (100) crystal planes of the COF material. Notably, the number of structural units in PVP, becomes a decisive factor in regulating this specific interaction, thereby achieving fine thickness control of the nanosheets while maintaining excellent dispersion. This strategy demonstrates high versatility, successfully applied not only to the preparation of imine‐linked 2D COF materials but also opening new avenues for the synthesis of other types of COFs, such as polyimide‐based 2D COFs, boron‐based 2D COFs, and imine‐linked 3D COFs. In addition to PVP, some surfactants can self‐organize into stable bilayer structures in water/ethanol solutions, where the hydrophobic alkyl chains aggregate inside the bilayer and the polar heads of the surfactants are located on the outer surfaces facing the aqueous solution.^[^ ^67^ ^]^ This 2D bilayer structure provides an ideal platform for constructing COF nanosheets on its outer surface.^[^ ^68^ ^]^ Liu et al.^[^ ^69^ ^]^ developed a surfactant‐directed growth supramolecular mineralization strategy, in which precursors undergo 2D directional pre‐assembly at the layered micellar interface formed by the fluorinated surfactant perfluorooctadecanoic acid. Subsequent 2D supramolecular assembly of monomers can directly grow and crystallize into 2D COF nanosheets with diameters of several micrometers.

Recently, Feng's group^[^ ^70^ ^]^ have achieved notable advancements in exploring beyond traditional water‐air interfacial chemistry (Figure 4b). They innovatively employed micellar systems to mimic the chemical environment of water surfaces, meticulously regulating the self‐assembly behavior of charged surfactants, including anionic SOS and cationic surfactants cetyltrimethylammonium bromide (CTAB), to surpass the critical micelle concentration threshold. Consequently, they constructed micellar supramolecular structures. In these structures, the hydrophobic cores simulate an air‐like nonpolar environment, while the water layers formed around the hydrophilic head groups of the surfactants serve as interfacial roles. Porphyrin molecules pre‐arrange into J‐aggregate morphologies on the micelle surface, a process actively induced by the charged head groups of the micelles, forming a confined geometric structure with a defined orientation that significantly enhances the reactivity and selectivity of the micelle surface. This innovative approach has demonstrated high selectivity (up to 99% and above) and exceptional yields (no less than 92%) in various reversible and irreversible chemical reactions. Furthermore, this strategy has successfully synthesized 2D polyimine and 2D polybenzimidazole materials, with the polymer thin layers exhibiting uniform circular nanosheet morphologies, ≈2.0 µm in diameter, and possessing thicknesses of 15 and 18 nm, respectively. This achievement not only enriches the application scope of water surface chemistry but also significantly broadens its accessibility and practicality in complex chemical synthesis fields.

Beyond nanosheets, in recent, Cooper et al.^[^ ^71^ ^]^ has unveiled two types of nanoscale COFs photocatalysts, synthesized through a bottom‐up approach facilitated by the CTAB and SDS. These COFs exhibit unique morphologies as nanobelts and nanodisks, respectively. Their preparation involves the application of ultrasonic technology under environmentally benign conditions. This innovative methodology not only streamlines the operational procedure but also substantially advances the mass production and scalability of nanoscale COFs. Compared to their bulk counterparts, the strategy of modulating COF crystal size via surfactants notably enhances the aqueous dispersion properties and light‐harvesting efficiency of the products, thereby opening up new avenues for the application of COFs in the field of photocatalysis.

#### 2.2.2 Nanofibers

While surfactants can be readily removed through washing, the assembly of soft templates becomes particularly challenging when synthesizing COFs via organic phases under harsh conditions. To address this issue, Wang et al.^[^ ^72^ ^]^ designed a strategy combining surfactants with acid regulation to meticulously shape the morphology of β‐ketoamine‐linked COF through a solvothermal synthesis process. In this process, surfactants acting as stabilizers pre‐encapsulated monomers and oligomers to form ordered micellar structures, guiding the growth of TpPa in a fibrous morphology rather than irregular aggregates. Notably, they also found that, in addition to surfactants, acetic acid played an indispensable role in morphology regulation. This is because the amino groups within the oligomers could be precisely protonated under acid regulation, influencing the maturation stage of TpPa's specific morphology formation and thus enabling fine control over its growth pattern. The stabilizing effect of surfactants combined with the precise control of acid regulation resulted in TpPa nanofibers with diameters of ≈20 nm and lengths reaching several micrometers. This achievement not only reveals the intrinsic mechanisms of morphology formation and evolution in TpPa‐COFs but also provides valuable insights into surfactant‐assisted fine‐controlled synthesis, guiding the development of COFs for practical applications. Furthermore, Li et al.^[^ ^73^ ^]^ employed a surfactant‐mediated solvothermal synthesis strategy to prepare TFP‐PPDA COF materials with excellent morphological uniformity and high crystallinity. CTAB, serving as the surfactant, regulated the specific surface area and structural order of the COF materials. Experimental results demonstrated that the introduction of CTAB significantly altered the microstructure of TFP‐PPDA COFs, transitioning them from a traditional bulk porous morphology to a fluffy, long‐threaded twisted structure. This transformation not only enhanced pore connectivity but also greatly facilitated the diffusion efficiency of guest molecules within the material. Compared to the traditional solvothermal method without surfactants, the specific surface area of TFP‐PPDA COFs synthesized with CTAB assistance exhibited a substantial increase, jumping from 291 to 705 m^2^ g^−1^. This discovery holds significant implications for enhancing the application potential of COF materials in gas adsorption, catalytic reactions, energy storage, and other fields.

#### 2.2.3 Nanospheres/Nanovesicles

Surfactant‐assisted multiphase solvent method provides a new perspective for morphological control of COFs.^[^ ^17^ ^]^ Beyond the aforementioned 1D and 2D morphologies of COFs, Zhao et al.^[^ ^74^ ^]^ innovatively introduced emulsion interface polymerization technology. They selected the surfactant dodecyltrimethylammonium bromide (DTAB) as an efficient emulsifier and utilized a biphasic solvent system (a mixture of water and n‐butanol) to induce and control the nucleation and growth process of COFs. This strategy facilitated preferential nucleation of COFs at the biphasic interface, followed by gradual development into hollow‐structured COF microspheres within the confined space. The synthesized H‐COF‐OMe exhibited perfect hollow spherical morphology with smoother surfaces, uniform particle sizes, and good dispersion. The uniqueness of this method lies in its precise regulation of the multiphase solvent interface, which not only achieved the construction of hollow spherical micro/nanostructures in COF materials but also significantly increased their specific surface area and introduced abundant structural defect interfaces. These characteristics are of great significance for enhancing the functionality and application potential of the materials.

Certain surfactants can further optimize the synthesis of COFs by altering the properties of the reaction medium, such as polarity and electrical properties, thereby providing the most suitable reaction environment.^[^ ^76^ ^]^ This not only aids in the preparation of high‐quality COF materials but also reduces synthesis costs and improves production efficiency. Polyethylene glyco‐400 (PEG‐400) as a non‐ionic surfactant, has been widely used as a pore‐forming agent for structurally controlled silica and as a modifier for polymers.^[^ ^77^, ^78^ ^]^ Qiu et al.^[^ ^79^ ^]^ ingeniously designed a mixed solution system based on water and PEG‐400, which exhibits low viscosity and high homogeneity. Serving as a mild and environmentally friendly reaction medium, this system enables efficient synthesis of spherical COFs with excellent crystallinity, morphology, and stability under near‐room temperature conditions in an open environment (without inert gas protection and at atmospheric pressure). By finely tuning the solvent ratio and introducing chemical modulators such as monoaldehydes or monoamines, the team also synthesized three other COFs with distinct morphologies (fibrous, urchin‐like, and flower‐like), demonstrating the high flexibility and effectiveness of this method in morphological control. Furthermore, the study evaluated the versatility of this green reaction system in the synthesis of two other 2D COFs, providing strong support for the green construction of the COF material library. Additionally, preliminary mechanistic studies indicate that the polyethylene glycol‐based aqueous solution plays a role as a soft template in directing the formation of COF morphologies, opening up new perspectives and strategies for the future development of more morphologically controlled and high‐performance COF materials. Furthermore, polyethylene glycol diamine (molecular weight 2000) as a non‐ionic surfactant has also been utilized in the synthesis of COF nanospheres.^[^ ^80^ ^]^

**Figure 5:**
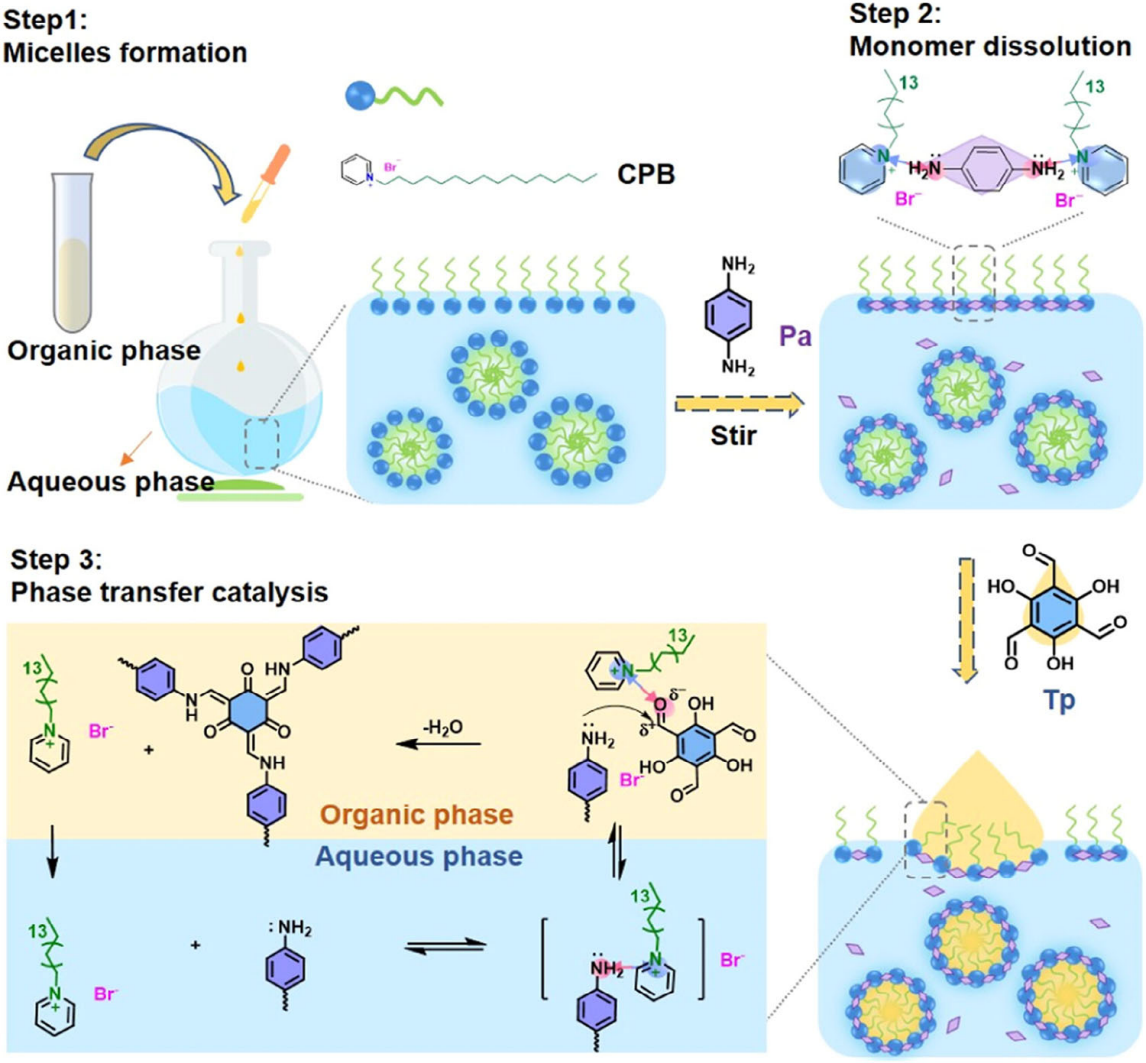
Synthesis schematic diagram of TpPa‐COF via surfactant‐assisted emulsion polymerization phase transfer catalysis route. Reproduced with permission.^[^ ^75^ ^]^ Copyright 2023, American Chemical Society.

In addition to serving as templates for controlling polymer morphology under mild conditions, certain surfactants, such as pyridinium‐based cationic surfactants, also exhibit catalytic effects during the synthesis of COFs. They can accelerate the polymerization reaction between monomers and improve the synthesis efficiency of COFs. Jin et al.^[^ ^75^ ^]^ proposed an innovative strategy for synthesizing β‐ketoamine‐linked COFs through emulsion polymerization under phase‐transfer catalysis (**Figure** **5**). The core of this method lies in the use of commercially available pyridinium‐based surfactants, which not only stabilize the emulsion system as emulsifiers but also function as efficient catalysts and morphology modifiers. By finely tuning the interactions at the emulsion interface, the researchers were able to prepare TpPa‐COF with different morphologies, including spheres, vesicles/bowls, and fibers. Furthermore, the obtained COF emulsion can be directly converted into thin films by applying an electric field, providing a novel and simple route for the preparation of COF thin films. This phase‐transfer catalysis method also demonstrated its potential for large‐scale synthesis, successfully synthesizing gram‐scale quantities of TpPa‐COF. More importantly, this method offers significant environmental advantages by avoiding the use of acidic catalysts and large amounts of organic solvents, and the reaction can be completed in just 10 min in an oil‐water system at room temperature. It achieves an efficient and environmentally friendly synthesis process and demonstrates its potential in large‐scale production and thin‐film preparation.

### 2.3 Controlling Structure of COFs

The control over the structure of COFs, particularly their pore size, chirality, and single‐crystal formation, is paramount in dictating their functional properties and applications.^[^ ^81^, ^82^ ^]^ Surfactants play a pivotal role in this process, offering unique advantages in templating, guiding, and stabilizing the COF structure. By meticulously tuning the type, concentration, and interaction mechanisms of surfactants, researchers can achieve a fine degree of control over the pore hierarchy, chiral induction, and crystallization behavior of COFs. The precise mechanism by which surfactants influence these structural parameters is of great interest and forms the basis for numerous recent advancements. This section highlights the crucial role of surfactants in structural control, as exemplified in various studies.

#### 2.3.1 Pore Size

**Figure 6:**
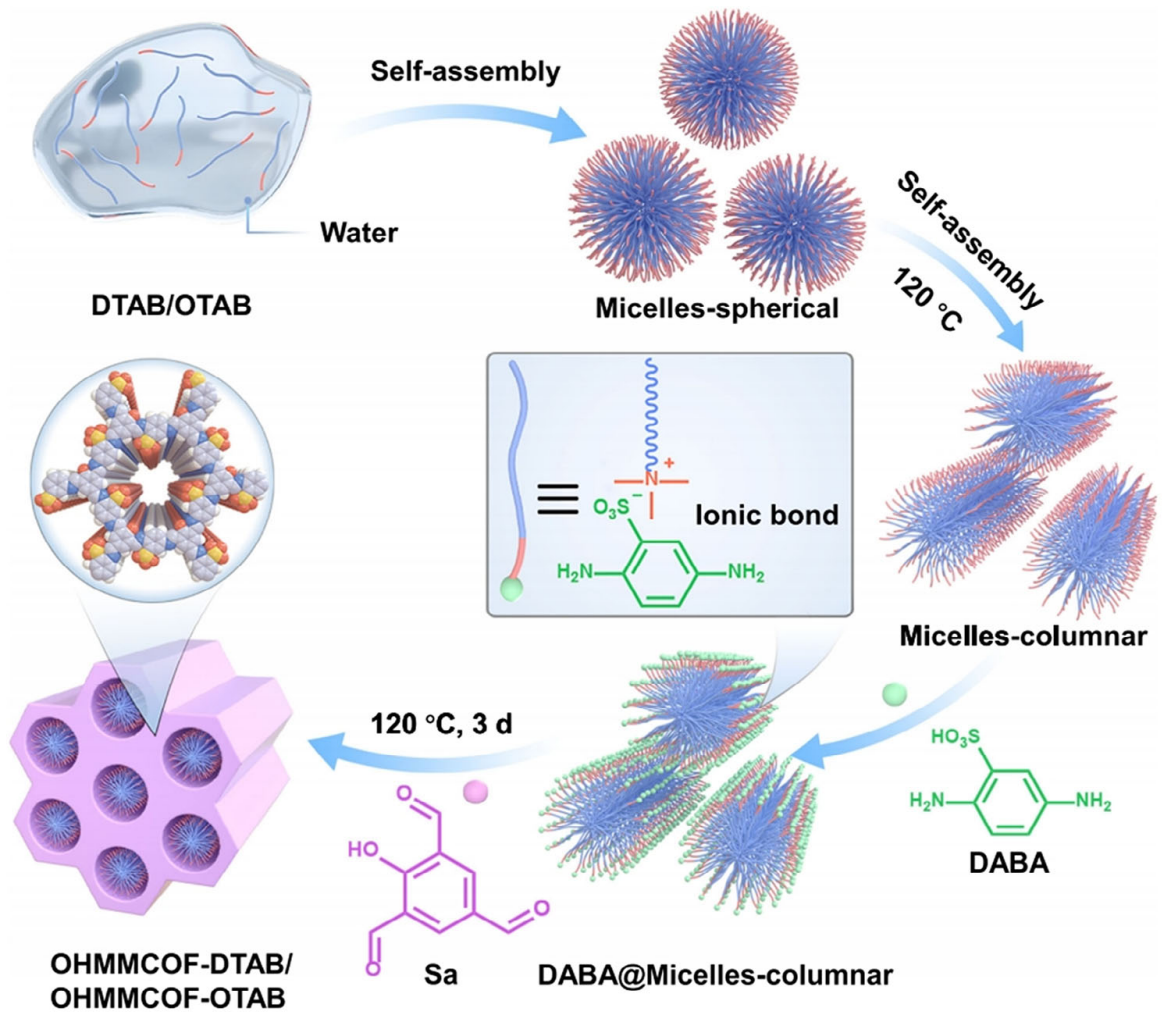
Schematic diagram of surfactant soft‐template‐directed synthesis of ordered hierarchical microporous/mesoporous COFs. Reproduced with permission.^[^ ^92^ ^]^ Copyright 2024, Springer Nature.

Due to the rigidity and size constraints of building blocks, most existing COFs are microporous (pore size < 2 nm), limiting the accessibility of functional groups and thus their full potential in practical applications.^[^ ^83^, ^84^ ^]^ In contrast, hierarchical porous COFs with mesoporous structures typically exhibit reduced diffusion resistance for small guest molecules, easily accessible active sites, and significantly improved mass transfer.^[^ ^85^, ^86^ ^]^ Although surfactant‐based soft‐template‐directed assembly is widely used for the construction of mesoporous structures in aluminosilicates, silicon dioxide, metal oxides, and other polymer,^[^ ^87^, ^88^, ^89^ ^]^ achieving ordered hierarchical porous COFs through soft‐templating remains a formidable challenge.^[^ ^90^ ^]^ The crux of this challenge lies in the fact that soft‐templating methods typically rely on specific hydrophobic interactions of amphoteric surfactants in aqueous phases to form micellar soft templates.^[^ ^91^ ^]^ However, most COF synthesis processes employ organic solvents and generally require harsh synthetic conditions (high temperatures, long reaction times, vacuum environments). Additionally, the unique reversible covalent bond properties of COFs add complexity to the introduction and subsequent removal of templates. Ma et al.^[^ ^92^ ^]^ proposed an innovative method (**Figure** **6**) by using water as the solvent and selecting two amphoteric surfactants with different alkyl chain lengths, DTAB and octadecyltrimethylammonium bromide (OTAB), as soft templates. The use of water as the solvent not only avoids the random distribution of amphoteric surfactants but also ensures their arrangement according to the expected hydrophobic self‐assembly mode. In water, when the surfactant concentration reaches a certain value, the hydrophobic groups of multiple surfactant molecules (or ions) interconnect, while the hydrophilic groups orient toward the aqueous phase, forming colloidal aggregates. By tightly binding the soft templates to the anionic COF backbone through ionic bonds, this method effectively prevents the templates from being excluded from the COF growth area due to strong crystallization driving forces. Consequently, this method successfully introduces an ordered arrangement of soft templates within the microporous COFs. After the completion of COF synthesis, the templates are removed through ion exchange while maintaining the high crystallinity of the COFs, ultimately yielding COFs with ordered microporous/mesoporous structures. Notably, the size and distribution of the mesopores can be flexibly adjusted by tuning the length, concentration, and usage ratio of the templates. This innovative soft‐templating strategy provides the possibility for synthesizing a variety of ordered hierarchical porous COFs, greatly enhancing the mass transfer efficiency and accessibility of functional groups in COFs.

#### 2.3.2 Chirality

In recent years, chiral COFs have demonstrated enticing application potential across multiple domains due to their unique chiral characteristics.^[^ ^93^, ^94^ ^]^ These applications span chiral recognition technologies, efficient chiral separation strategies, asymmetric catalytic platforms, and the development of chiral optical materials.^[^ ^95^, ^96^ ^]^ Inspired by the inducing role of amino acid‐based chiral surfactants in the formation of mesoporous silica,^[^ ^97^ ^]^ Zheng et al.^[^ ^98^ ^]^ innovatively extended this concept to the synthesis of chiral COFs in aqueous environments. They ingeniously applied amino acid derivative surfactants with adjustable chiral sites to this process, facilitating the effective transfer of chiral information through non‐covalent interactions such as hydrogen bonding and electrostatic interactions. Compared to traditional induction methods based on strong covalent bonds, this mechanism of weak interactions exhibits higher flexibility and environmental adaptability, enabling the construction and adjustment of chiral structures without the need for high activation energy. The study revealed subtle changes in hydrogen bonding and electrostatic interactions between the inducers and building blocks under different pH conditions, directly leading to phase transitions in chiral COFs and polarity inversion in the Cotton effect. The team also conducted a systematic investigation into the structural characteristics of amino acid derivative surfactants (e.g., hydrophobic chain length, headgroup type), providing an in‐depth analysis of their influence on the synthesis of chiral COFs. The weak interactions, including hydrogen bonding and electrostatic interactions between surfactants and precursors, simultaneously promoted the helical twist and self‐correction crystallization of the precursors. Ultimately, three types of COFs with 3D chiral imine chain structures were successfully synthesized, validating the broad applicability of this method. Subsequently, they utilized a similar strategy to synthesize 3D COFs with left‐handed and right‐handed fibrous shapes, achieving a conformational purity of up to 85%.^[^ ^99^ ^]^ These work open up a new pathway for the efficient construction of chiral COF materials from achiral precursors, significantly enriching the family of chiral COFs.

**Figure 7:**
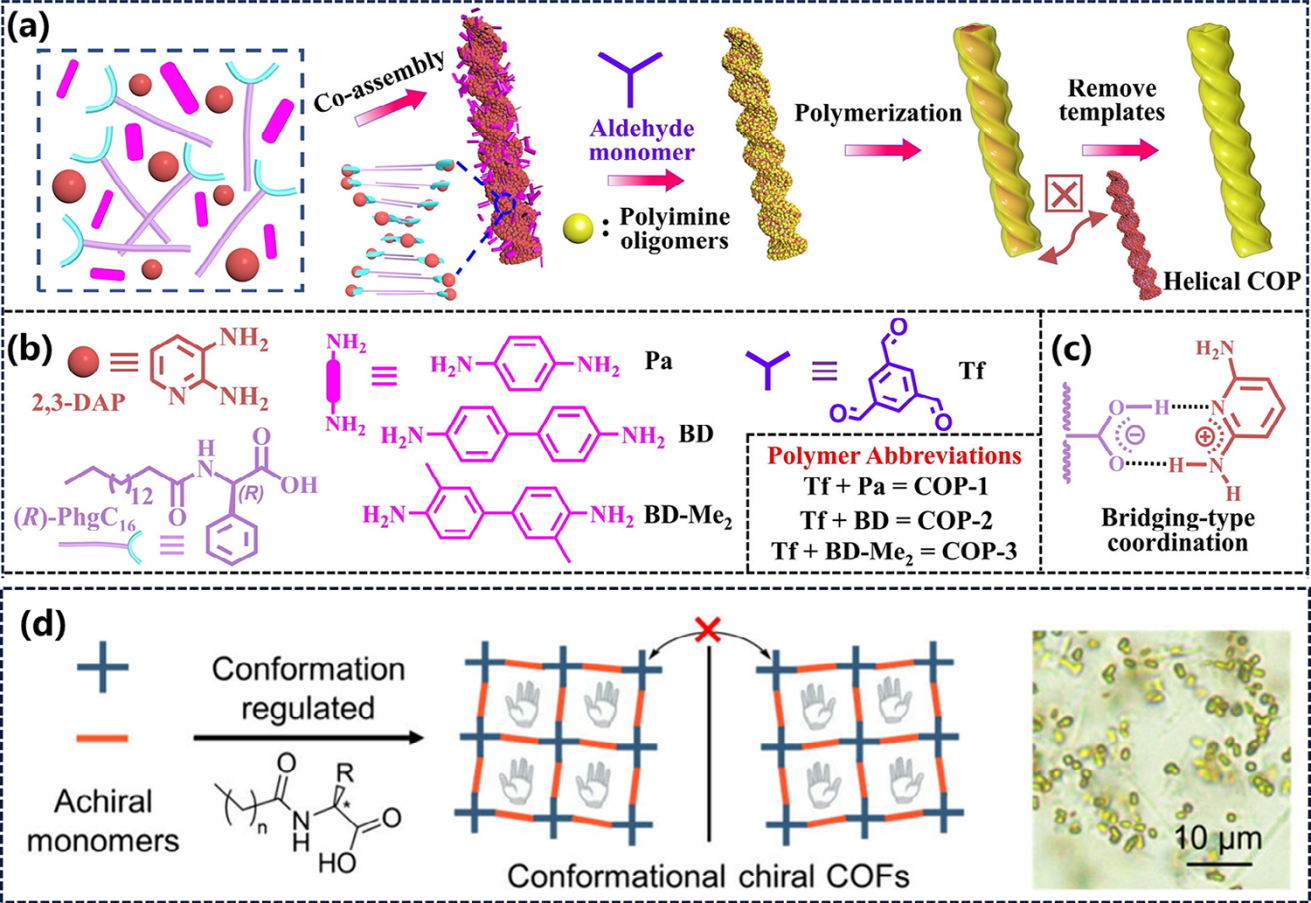
a) Schematic diagram of the synthesis of helical COF using 2,3‐DAP‐mediated chiral surfactant templating method. b) Molecular structures of the chemicals involved. c) Schematic representation of the formation of bridging hydrogen bonds between (R)‐surfactant and 2,3‐DAP. Reproduced with permission.^[^ ^100^ ^]^ Copyright 2023, Wiley‐VCH. d)Schematic diagram of chiral assisted synthesis with amino acid derivatives, and optical micrograph of the product. Reproduced with permission.^[^ ^101^ ^]^ Copyright 2024, American Chemical Society.

Inspired by the pivotal role of chiral molecules in constructing complex structures in nature,^[^ ^102^ ^]^ chiral surfactants, such as amino acids, serve as templates to impart specific spatial orientations and configurations during the fabrication of COFs, thereby leading to the formation of chiral COF structures. Wang et al.^[^ ^100^ ^]^ developed an innovative approach utilizing 2,3‐diaminopropionic acid (2,3‐DAP)‐mediated supramolecular templating was employed to prepare chiral imine‐linked polymer nanotubes constructed from achiral spatially symmetric monomers possessing mesoscopic helicity (**Figure** **7**a–c). The templating molecules, (R)‐ and (S)‐PhgC_16_, are amino acid derivatives obtained through the acylation of phenylglycine with hexadecyl chloride. Simply, first dissolve (R)/(S)‐PhgC_16_, 2,3‐DAP, and the active amine monomers in a suitable solvent. Then, add a poor solvent (water) under stirring to facilitate their chiral supramolecular co‐assembly. Next, add the active aldehyde monomer at room temperature to trigger the Schiff base reaction on the surface of the supramolecular assembly. After the reaction is complete, the solid product is filtered and collected, extracted in anhydrous ethanol, and the supramolecular template and imine oligomers are removed. Furthermore, Sun et al.^[^ ^101^ ^]^ proposed an additional synthetic method employing chiral amino acid derivatives as templates, successfully preparing highly crystalline chiral COFs (Figure 7d). In this study, COF‐300 was selected as the research model, and a meticulously designed synthetic pathway was utilized to condense L‐alanine with palmitoyl chloride to generate C_16_‐L‐AlaA as the crucial chiral template. These templates spontaneously formed dispersed chiral micelles in aqueous solution, which were then efficiently adsorbed onto the surfaces of COF crystals upon the addition of precursor materials and ultimately encapsulated within the pores. This process disrupted the original mesoscopic racemic structure of achiral COF‐300, successfully constructing single‐crystalline COFs with well‐defined conformational chirality. Notably, upon removal of the template molecules and water molecules, the structure of COF‐300 underwent transitions from an open conformation to a closed conformation and even a vacuum conformation. By precisely controlling the chain length, concentration, and configuration of the chiral templates, the research team achieved precise regulation of the stereochemical properties of the products, enabling flexible adjustment of circular dichroism signals across the ultraviolet to infrared spectral range. Additionally, they successfully synthesized chiral COF‐304, validating the broad applicability of this method. These above studies have deepened our understanding of chiral induction mechanisms and provided robust support for the customized synthesis of chiral COF materials and their exploration in various frontier applications.

#### 2.3.3 Single‐Crystal

**Figure 8:**
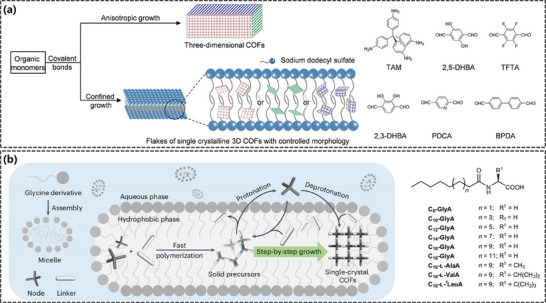
a) Schematic diagram of the synthesis of COF single crystals assisted by SDS lamellar structures. Reproduced with permission.^[^ ^126^ ^]^ Copyright 2023, American Chemical Society b) Schematic representation of the growth of single‐crystalline COFs within the hydrophobic compartments of surfactant micelles. Reproduced with permission.^[^ ^108^ ^]^ Copyright 2023, Springer Nature.

The synthesis of COFs often entails challenges such as high temperatures, vacuum operations, prolonged reaction times, and low solubility of the initial building blocks in conventional solvents, which collectively hinder the control over the size of COF crystallographic domains and crystal morphology.^[^ ^103^, ^104^ ^]^ The preparation of single‐crystalline COFs with controllable morphologies can enhance the elucidation of their local structures and deepen the understanding and application of structure‐property correlations.^[^ ^105^, ^106^ ^]^ To obtain pure phases, surfactant‐assisted strategies are highly desirable. Surfactants can adsorb onto the crystal surfaces during COF crystal growth, thereby influencing the crystal growth kinetics, such as crystal growth rates and morphologies.^[^ ^107^ ^]^ Beyond the aforementioned interfacial methods for obtaining single‐crystalline COF thin films, Zheng et al. innovatively employed SDS as a lamellar surfactant to effectively constrain the growth of 3D COFs in aqueous media, successfully preparing single crystalline flakes of 3D COFs nanosheets with five distinct morphologies, edge configurations, precursor crystal faces, and surface chemical properties that could be finely tuned (**Figure** **8**a). Amine and aldehyde monomers self‐assembled into single‐crystalline nanosheets within the lamellar structures of SDS in NaCl aqueous solution, as the adsorption of SDS selectively passivated specific crystal faces of the 3D COFs. The prepared single‐crystalline nanosheets exhibited excellent chemical stability, mechanical robustness, and thermal stability, making them ideal candidates for resolving fine structures (such as monomeric building blocks, edge fine structures, pore networks, grain boundary characteristics, and subtle lattice distortions within 3D COFs via AC‐HRTEM with an unprecedented spatial resolution of ∼1.7 Å. This provided robust experimental evidence for a deeper understanding of the microstructure‐property relationships of 3D COFs. They found that the type of surfactant (varying alkyl chain lengths and head groups) played a crucial role in modulating the crystallization process, with the interplay of surfactant order and hydrophobicity leading to differences in COF crystallinity and surface roughness. In another of their synthetic efforts targeting single‐crystalline COFs (Figure 8b),^[^ ^108^ ^]^ They used micelles formed from amphiphilic amino acid derivatives with long hydrophobic chains to prevent precipitation of materials resulting from the polymerization of monomers in water through weak interactions (hydrogen bonding, electrostatic interactions, and hydrophobic interactions). Similarly, they further discovered that various parameters, such as the hydrophobic chain length and head group of the amphiphilic amino acid derivatives, the addition of catalysts, reaction temperatures, and the cations coordinating with the amino acid derivatives, significantly influenced crystallization. This synthetic strategy was universal, enabling the production of one 2D and five 3D single‐crystalline COFs on the gram scale with yields of ≥92%.

### 2.4 Enhancing Processability of COFs

The practical application of COFs hinges on their ease of processing and integration into various material systems. Surfactants play a critical role in enhancing the processability of COFs by facilitating their dispersion, stabilization, and shaping into desired forms.^[^ ^109^ ^]^ Through the formation of colloidal solutions, stabilization of particles, and guidance of self‐assembly, surfactants enable the preparation of COFs with improved dispersibility, stability, and customizability. This section explores how surfactants contribute to the processability of COFs, enabling their potential use in advanced applications such as 3D printing and surface coating. By understanding the mechanisms underlying these processes, researchers can better harness the full potential of COFs in practical scenarios, as demonstrated in several recent studies.

#### 2.4.1 Colloid

**Figure 9:**
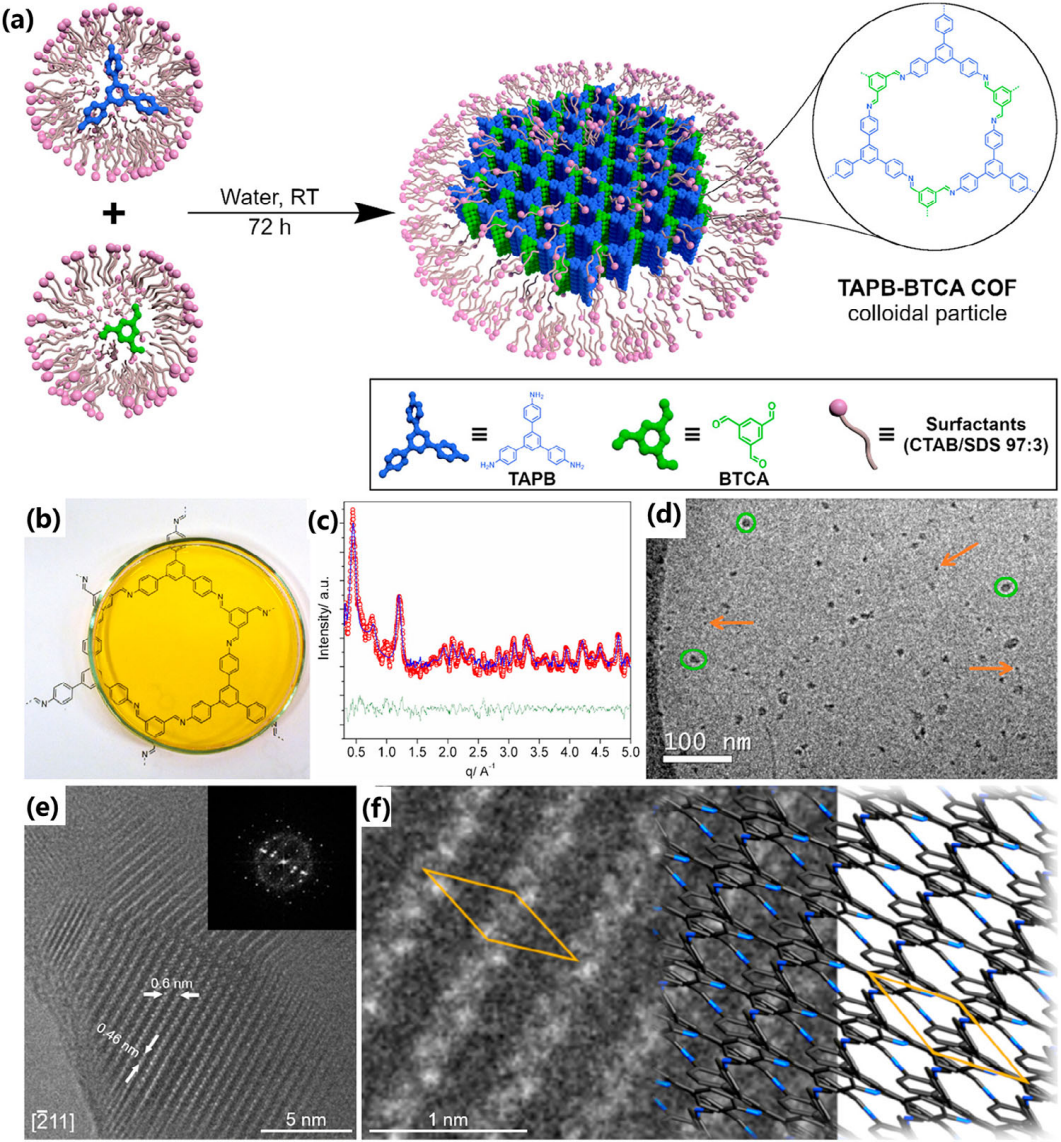
a) Schematic diagram of the surfactant‐assisted synthesis of COF nanoparticle colloids. b) Optical photograph, c) synchrotron X‐ray diffraction data, d) low‐temperature transmission electron microscope (TEM) image, e,f) magnified high‐resolution TEM (HR‐TEM) image and corresponding schematic diagram of the structural model of COF nanoparticle colloids. Reproduced with permission.^[^ ^112^ ^]^ Copyright 2020, American Chemical Society.

The synthesis of COFs often involves harsh conditions, resulting in products that are typically powdery and difficult to process, which limits the reprocessing and large‐scale application of COFs.^[^ ^110^, ^111^ ^]^ During the synthesis of COFs, components such as monomers and catalysts often struggle to disperse uniformly in water or other solvents. The presence of surfactants can effectively reduce the interfacial tension between these components, enabling better dispersion in solvents. Puigmartí‐Luis et al.^[^ ^112^ ^]^ proposed an innovative strategy by utilizing a mixture of cationic CTAB and SDS surfactants (CTAB/SDS 97:3) to form a cationic mixed micelle system, successfully preparing a stable and uniform colloidal solution of crystalline COF nanoparticles in the aqueous phase (**Figure** **9**). This not only overcame the challenge of poor solubility of COF molecular building blocks in the aqueous phase but also achieved effective regulation of COF nanoparticles. The resulting colloidal solution exhibited good stability and dispersion, enabling the processing of COFs into various 2D and 3D shapes. Furthermore, this colloidal solution could be used as a special “ink” to directly deposit COFs onto surfaces through printing technology, expanding the application range of COF materials. The Yang team^[^ ^113^ ^]^ continued and expanded upon this strategy and successfully synthesizing TP‐TTA‐COF colloids in the CTAB/SDS mixed micelle system. To further explore the application potential of their composites, the researchers uniformly dispersed commercial SiO_2_ nanoparticles (with an average particle size of ≈26 nm) in the TP‐TTA‐COF colloidal solution and prepared a series of composites with different COF layer thicknesses by regulating the number of TP‐TTA‐COF layers.

Surfactants can form a protective layer on the surface of COFs particles, preventing their mutual agglomeration and thereby enhancing the stability and processability of COFs. Jiang et al.^[^ ^114^ ^]^ have developed a novel and universal uniform nucleation strategy that precisely regulates and synthesizes highly uniform colloidal nanoparticles of COFs, further enabling controlled self‐assembly of these particles into superstructures in multiple dimensions. Specifically, they achieved fine control over the size of COF colloidal particles by adjusting the concentrations of acetic acidcatalyst and PVP surfactant in the reaction system. Notably, the introduction of PVP not only optimized the dispersion performance of the particles, effectively preventing agglomeration between them, but also created favorable conditions for subsequent multi‐level self‐assembly of the particles in 1D linear, 2D planar, and even 2D spaces, thereby constructing a diversified system of superstructures. This research provides new perspectives and strategies for the controlled synthesis and advanced structural construction of COF materials. Furthermore, surfactants exhibit excellent dispersion and stabilizing effects, preventing the agglomeration and precipitation of COFs particles during synthesis. Zhou et al.^[^ ^115^ ^]^ found that SDS can improve the aggregation of β‐ketamine COFs (TPAQ‐COFs) after synthesis, inhibiting their stacking and enhancing their adsorption performance. Additionally, based on work on synthesizing ≈10 nm conjugated network oligomers utilized dual surfactant assistance,^[^ ^116^, ^117^, ^118^ ^]^ Qiao and his team successfully prepare two stable colloidal dispersions with particle sizes less than 10 nanometers using *bis*‐dibenzothiazole diamine and *tri‐*benzothiazole triamine as basic building units in a co‐condensation reaction with triformylphloroglucinol, assisted by cationic micelles (specifically a mixed system of CTAB and SDS).^[^ ^119^ ^]^ These obtained colloidal crystals exhibited properties similar to printing inks, endowing them with potential for large‐scale manufacturing of panel‐type photoelectrode films with smooth and uniform surface characteristics. The above advancements provide new ideas and methods for the synthesis and processing of COF materials, which are of great significance for promoting the development of porous material science.

#### 2.4.2 3D printing

**Figure 10:**
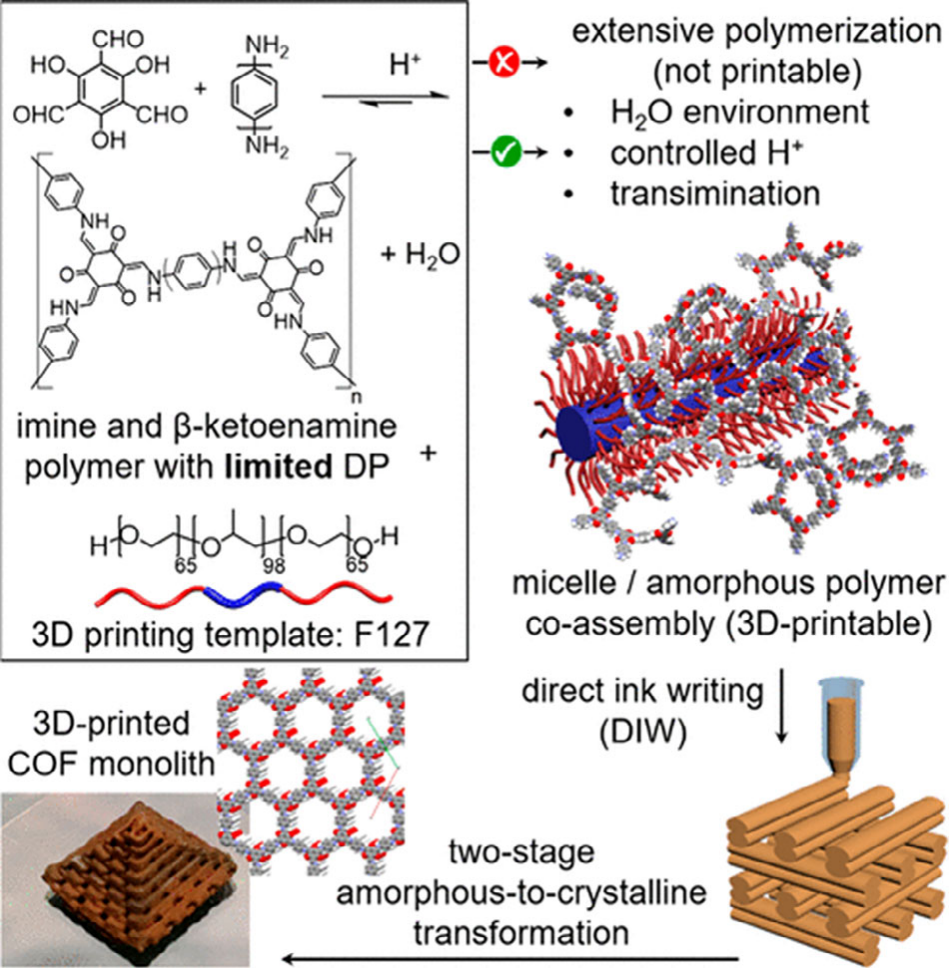
a)Schematic diagram for the synthesis of 3D printed hierarchical porous COFs through supramolecular template co‐assembly. Reproduced with permission.^[^ ^125^ ^]^ Copyright 2019, American Chemical Society.

3D printing technology has opened up new manufacturing pathways for complex structures that are difficult to achieve with traditional manufacturing methods, significantly enhancing design freedom and processing capabilities.^[^ ^120^, ^121^ ^]^ For COFs materials, this technology enables precise control and high customization of their shapes, thereby enhancing their performance characteristics and greatly expanding their application scope.^[^ ^122^, ^123^ ^]^ Polymer additives such as Pluronic F127 are often mixed with active materials to ensure the rheological properties of the ink and strong interactions between particles.^[^ ^124^ ^]^ Ke et al.^[^ ^125^ ^]^ innovatively adopted a strategy using the amphiphilic surfactant Pluronic F127 as a 3D printing template to guide the ordered assembly of imine polymer precursors in an aqueous environment (**Figure** **10**). By introducing an excess water environment combined with a precisely controlled dose of acid catalyst, they effectively regulated the rate and extent of imine condensation during COF formation, thereby achieving the co‐assembly of amorphous imine polymers into 3D printed hydrogels. After the printing process, further heat treatment of the amorphous polymers induced their polymerization and reconstruction of imine network, achieving a transition from an amorphous state to a highly crystalline state. Finally, by removing F127, various COFs based on β‐ketoamine and imine groups were prepared. These materials exhibited high crystallinity, ultra‐large specific surface areas, and well‐defined porous structures, while maintaining excellent structural integrity and significant mechanical strength. This approach pioneers a new pathway for the preparation of complex 2D macroscopic porous structures of COF materials. Furthermore, Zamora et al.^[^ ^122^ ^]^ optimized the conversion process of colloidal suspensions into fully 3D printable COF‐based inks. They innovatively designed a microfluidic system that could finely regulate the gelation process of COF inks at the printing nozzle, enabling precise layer‐by‐layer construction of materials. This breakthrough made it possible to directly output large‐scale, binder‐free COF structures from digital models without relying on high‐temperature and high‐pressure conditions or toxic organic solvents, significantly promoting the green development of the COF processing field.

## 3 Summary and Prospect

In recent years, surfactants have demonstrated significant advantages in the preparation of COFs, becoming an important research direction in this field. The introduction of surfactants not only mediates the assembly behavior of monomers through their unique molecular structures and properties, enabling precise control over the morphology, pore structure, and crystallinity of COFs, but also significantly improves the synthesis efficiency and processability of COFs, laying the foundation for functional applications. However, despite the broad application prospects and remarkable achievements of surfactants in COF preparation, current research and practice still inevitably face several challenges and deficiencies that need to be addressed urgently:

The interfacial synthesis of COF films represents a transformative approach to achieving large‐area, ordered architectures with tailored functionalities. While surfactant‐mediated strategies at gas‐liquid and liquid‐liquid interfaces have enabled breakthroughs in crystallinity and film uniformity, surfactant‐assisted solid‐liquid interfacial polymerization remains unexplored, the surfactants could mediate monomer adsorption on solid substrates (e.g., graphene or oxides) via electrostatic/hydrophobic interactions. This may enable epitaxial growth of COFs with orientation dictated by the substrate lattice, offering a pathway to hybrid materials for electronics or catalysis. Moreover, critical gaps remain in bridging laboratory‐scale successes to industrial viability. Current methods often prioritize structural perfection over mechanical robustness, leading to fragile films prone to cracking under stress. A key challenge lies in reconciling the dynamic nature of interfacial reactions with the need for scalable, defect‐tolerant fabrication. Future advancements should focus on integrating stimuli‐responsive surfactants that adapt to polymerization kinetics, thereby enabling in situ repair of grain boundaries. Additionally, the development of hybrid surfactants—combining templating, stabilizing, and functionalizing roles—could streamline the synthesis of multifunctional films for flexible electronics or ion‐selective membranes. Leveraging advanced characterization tools, such as in situ atomic force microscopy, to map surfactant‐monomer interactions in real time will deepen mechanistic understanding and guide the rational design of next‐generation COF films. By addressing these aspects, interfacial synthesis could evolve from a niche technique to a cornerstone of COF‐based device manufacturing.

Morphological engineering of COFs via surfactants has unlocked unprecedented control over their physical and chemical properties, yet this field remains largely empirical. While template‐driven strategies yield diverse nanostructures (from nanosheets to hollow spheres) the lack of predictive models limits precision in achieving target morphologies. A central issue is the competing roles of surfactant self‐assembly and COF crystallization kinetics, which often result in trade‐offs between shape fidelity and crystallinity. To transcend these limitations, we advocate for a paradigm shift toward dynamic templating, where surfactants with tunable aggregation states (e.g., light‐ or redox‐responsive amphiphiles) guide morphology evolution during synthesis. Coupling this with machine learning frameworks to correlate surfactant properties (e.g., chain length) with final morphologies could democratize the design of bespoke COF architectures. Furthermore, integrating operando spectroscopic techniques, such as grazing‐incidence X‐ray scattering, would illuminate nucleation pathways and inform surfactant selection. By merging computational insights with adaptive surfactants, researchers can move beyond trial‐and‐error approaches, paving the way for COFs with programmable morphologies optimized for catalysis, sensing, or energy storage.

The precise control over COF structures (spanning pore architecture, chirality, and crystallographic perfection) is pivotal for unlocking their full functional potential. Nowadays, significant strides have been made in templating hierarchical porosity and inducing chirality via surfactants. While significant strides have been made in templating hierarchical porosity and inducing chirality via surfactants, fundamental challenges persist. For instance, current soft‐templating strategies often lack dynamic adaptability, limiting the synthesis of stimuli‐responsive COFs with tunable pore geometries. Future efforts should focus on integrating dynamic surfactants (e.g., pH‐ or redox‐responsive amphiphiles) to achieve real‐time structural reconfiguration. Additionally, the interplay between surfactant self‐assembly and COF crystallization kinetics remains underexplored; advanced computational tools, such as machine learning‐guided molecular dynamics simulations, could unravel these interactions and accelerate the design of COFs with user‐defined topologies. Furthermore, the translation of chiral induction mechanisms from small‐molecule systems to extended frameworks demands deeper mechanistic studies, particularly in understanding how weak non‐covalent forces propagate long‐range order. By addressing these gaps, surfactant‐assisted structural engineering could pave the way for next‐generation COFs tailored for asymmetric catalysis or molecular recognition.

The transition of COFs from laboratory curiosities to industrial applications hinges on their processability, yet this remains a formidable bottleneck. While surfactants have enabled colloidal dispersions and 3D‐printable inks, critical limitations must be overcome, such as scalability, surfactant residue, and ink stability. We propose that future research prioritize the development of multifunctional surfactants capable of dual roles (e.g., templating and crosslinking) to simplify post‐synthetic purification. For instance, surfactants with thermally cleavable bonds could template COF growth and then degrade upon mild heating, eliminating residues. Moreover, the integration of COF colloids into hybrid systems (e.g., with conductive polymers or inorganic nanoparticles) via surfactant‐mediated interfacial engineering could yield multifunctional composites for flexible electronics or energy devices. Another promising avenue lies in leveraging bio‐inspired surfactants (e.g., peptide amphiphiles) to mimic natural self‐assembly processes, enabling eco‐friendly fabrication of COF‐based membranes or sensors. By marrying surfactant innovation with advanced manufacturing techniques, the field can transcend current limitations and realize COFs as versatile, scalable functional materials.

The synergistic interplay between interfacial engineering, morphological control, structural precision, and processability defines the frontier of surfactant‐assisted COF construction. The interfacial assembly dynamics inherently govern morphological evolution. For instance, the Marangoni flows generated during gas‐liquid interfacial polymerization can align surfactant templates into anisotropic patterns, which subsequently template the growth of COF nanofibers with preferred orientations—a phenomenon bridging interfacial hydrodynamics to mesoscale morphology. Conversely, the crystallographic registry established during structural control feeds back into interfacial stability by modulating surfactant adsorption energies through lattice‐matching effects. Moreover, the structural hierarchy, from molecular pores to macroscopic architectures, exhibits multiscale interdependencies. The mechanical resilience required for 3D printing COF inks emerges from the synergy between surfactant‐mediated π‐π stacking gradients (structural control) and shear‐induced alignment of nanosheet building blocks (morphological engineering). This cross‐scale integration enables programmable mechanical properties through surfactant selection. Addressing these cross‐dimensional challenges demands a paradigm shift from isolated parameter optimization to system‐level design. Such integrated approaches will accelerate the development of COF materials where interfacial precision, morphological complexity, structural fidelity, and manufacturability are no longer competing objectives but mutually reinforcing attributes.

## Conflict of Interest

The authors declare no conflict of interest.
